# Exploring the decolorization efficiency and biodegradation mechanisms of different functional textile azo dyes by *Streptomyces albidoflavus* 3MGH

**DOI:** 10.1186/s12866-024-03347-9

**Published:** 2024-06-14

**Authors:** Mohamed E. El Awady, Fatma N. El-Shall, Ghada E. Mohamed, Ahmed M. Abd-Elaziz, Mohamed O. Abdel-Monem, Mervat G. Hassan

**Affiliations:** 1 Microbial Biotechnology Department, Biotechnology Research Institute, National Research Centre, El- Buhouth St. 33, Dokki, Cairo, Egypt; 2 Dyeing, Printing and Textile Auxiliary Department, National Research Centre, El-Buhouth St. 33, Dokki, Cairo 12622 Egypt; 3 Botany and Microbiology Department, Faculty of Science, Benha University, Benha, 13518 Egypt; 4 Molecular Biology Department, Biotechnology Research Institute, National Research Centre, El-Buhouth St. 33, Dokki, Cairo, Egypt

**Keywords:** *Streptomyces*, Azo dyes, Decolorization, Biodegradation, GC-MS, HPLC

## Abstract

Efficiently mitigating and managing environmental pollution caused by the improper disposal of dyes and effluents from the textile industry is of great importance. This study evaluated the effectiveness of *Streptomyces albidoflavus* 3MGH in decolorizing and degrading three different azo dyes, namely Reactive Orange 122 (RO 122), Direct Blue 15 (DB 15), and Direct Black 38 (DB 38). Various analytical techniques, such as Fourier Transform Infrared (FTIR) spectroscopy, High-Performance Liquid Chromatography (HPLC), and Gas Chromatography-Mass Spectrometry (GC-MS) were used to analyze the degraded byproducts of the dyes. *S. albidoflavus* 3MGH demonstrated a strong capability to decolorize RO 122, DB 15, and DB 38, achieving up to 60.74%, 61.38%, and 53.43% decolorization within 5 days at a concentration of 0.3 g/L, respectively. The optimal conditions for the maximum decolorization of these azo dyes were found to be a temperature of 35 °C, a pH of 6, sucrose as a carbon source, and beef extract as a nitrogen source. Additionally, after optimization of the decolorization process, treatment with *S. albidoflavus* 3MGH resulted in significant reductions of 94.4%, 86.3%, and 68.2% in the total organic carbon of RO 122, DB 15, and DB 38, respectively. After the treatment process, we found the specific activity of the laccase enzyme, one of the mediating enzymes of the degradation mechanism, to be 5.96 U/mg. FT-IR spectroscopy analysis of the degraded metabolites showed specific changes and shifts in peaks compared to the control samples. GC-MS analysis revealed the presence of metabolites such as benzene, biphenyl, and naphthalene derivatives. Overall, this study demonstrated the potential of *S. albidoflavus* 3MGH for the effective decolorization and degradation of different azo dyes. The findings were validated through various analytical techniques, shedding light on the biodegradation mechanism employed by this strain.

**Supplementary Information:**

The online version contains supplementary material available at 10.1186/s12866-024-03347-9.

## Introduction

Azo dyes have emerged as the predominant class of synthetic dyes [1, 2] due to their extensive utilization in diverse industries, particularly the textile sector. This preference stems from their cost-effectiveness, ease of production, and versatility for various applications [3]. However, wastewater from industries such as plastics, textiles, paper and leather printing contains large amounts of one or more synthetic azo bonds. These chemicals poses significant ecological hazards, including carcinogenic and mutagenic effects [4]. Among these risks, the discharge of dye effluents into natural water bodies has recently gained considerable public attention [1, 5, 6].

The environmental impact of azo dye waste is detrimental to ecosystems. Approximately 7 × 10^5^ tons of colorants are produced every year, with azo dyes that contain the –N = N– bond (azo) making up nearly 70% of the overall dyestuff production [7–9]. Water pollution caused by these dyes can inflict harm upon the aquatic ecosystem, leading to diminished light penetration that adversely affects organisms living in water. Consequently, this disruption hampers the vital process of photosynthesis [10]. Moreover, the toxic and carcinogenic properties of azo dyes pose risks not only to humans but also to other living organisms [11].

Reactive azo dyes are the most consumable type within the dye industry due to their high binding ability with cotton and cellulosic fibers, broad spectrum of colors, and color stability [12–14]. There are different types of reactive dyes; however, the common ones are characterized by the presence of chromophore groups such as azo and anthraquinone and reactive groups such as chlorotriazinile and sulphato-ethyl sulphonyl groups [15]. RO 122 is an anionic mono azo dye characterized by having monochlorotriazine and vinyl sulphone reactive groups [16]. It is widely used in dyeing, especially for silk and cotton, due to its high efficiency [17, 18]. It is commonly found in the effluents of textile industries as a calcitrant residue, making it highly resistant to conventional treatment methods [16, 19, 20]. Another reactive anionic azo dye is DB15, which is a benzidine based azo dye containing a benzidine group attached to other substituents by the diazo linkage [21, 22], characterized by its high affinity to cellulosic fibers. It exists as a microcrystalline powder with a purple to dark blue color. It also, called Sky Blue A and Direct Sky Blue 5B [23–25]. Similar to DB 15, DB 38 is a benzidine-based azo dye with three azo linkages [26] widely used in the dyeing industry for fabric, leather, cotton, cellulose materials, and plastic [27]. Also, these azo dyes possess mutagenic and carcinogenic properties for human beings and other live organisms, including plant and aquatic ones [28, 29]. Several publications have emphasized the crucial need to eliminate and degrade azo dyes in the environment, particularly in water, which serves as a vital resource for all living organisms. Addressing this pressing issue involves employing three main categories of technologies aimed at removing azo dyes from the environment: chemical, physical, and biological methods [10, 30–37]. However, the chemical and physical treatment approaches possess inherent limitations, including low efficiency, substantial sludge generation, high energy requirements, and significant costs [9, 38, 39].

Consequently, there is a critical need to enhance the philosophy of decolorization. Biological methods are the best way to get rid of azo dye pollution because they don’t create toxic waste or sludge and are cheaper than physical and chemical methods [40–42]. In biological treatment, microorganisms are pivotal in employing their metabolic pathways to eliminate pollutants. This process is facilitated by a variety of enzymes that can degrade the (–N = N–) bond, yielding aromatic amines as intermediate compounds. These amines can then be subsequently broken down by microorganisms, either aerobically or anaerobically [2]. This process typically involves the transfer of electrons from the pollutant molecule to the enzyme’s active site, where the oxidation reaction occurs. The enzyme then catalyzes the conversion of the pollutant into intermediate compounds, such as aromatic amines, which are often less harmful than the original pollutant. These intermediate compounds can subsequently undergo further degradation by microorganisms, either in aerobic or anaerobic conditions. Laccases, as multi-copper oxidases, are widely utilized for oxidizing dye by-products, either directly or through mediators such as 2, 2’-azino-bis-(3-ethylbenzothiazoline-6-sulfonic acid) (ABTS), facilitating the decolorization of textile dyes [43–45]. Microorganisms capable of decolorizing dyes have been discovered and examined in various environments, such as dye-polluted soil, water, sludge, and industrial wastewater [7, 39, 42]. Recent studies have extensively explored the use of microorganisms and higher plants in wastewater treatment to effectively remove azo dyes. These studies encompass various organisms, including bacteria [46, 47], algae [48], actinomycetes [28], fungi [49, 50], yeast [51], and higher plants [2, 52].

Time, temperature, pH, and dye concentrations are some of the factors that affect how well microbial cells remove dye color [28]. Actinobacteria, which are Gram-positive bacteria, are widely distributed in the environment, particularly in soil, and are known for their diversity and adaptability to harsh environmental conditions [29]. They have gained recognition for their ability to produce bioactive molecules and their capacity to degrade various substances. They are particularly well-suited for treating textile effluents due to their stability in different environmental conditions and their tolerance to toxic metal-containing textile dyes [53]. Research has shown that actinobacteria can degrade azo dyes through various mechanisms. For example, *Streptomyces*, a type of actinobacteria, makes extracellular peroxidases that help break down lignin into water-soluble polymeric compounds [54]. *Streptomyces* strains DJP1 and DJP2 have demonstrated potential in the discoloration of azo orange dye after 48 h of incubation [55]. *Streptomyces pactum* has shown the ability to discolorize dyes such as methyl red, HER navy blue, HB reactive magenta, and 3R orange, with oxidative biodegradation occurring through the action of enzymes like lignin peroxidase [56]. Additionally, *Streptomyces coelicolor* strain SPR7 has exhibited the ability to discolorize azo dyes including methyl red, methylene blue, orange green, metanil yellow, mordant black, and Sandopel blue P [54].

In the current study, RO 122, DB 15, and DB 38 were chosen due to their wide usage as colorants for different fabrics. So, the main objective of the present study was to check the ability of an actinobacterial strain obtained from Egyptian soil. This strain demonstrated the ability to decolorize and degrade three different azo dyes (RO 122, DB 15, and DB 38). Also, the study aimed to optimize various experimental parameters, including temperature, pH, initial dye concentration, carbon source, and nitrogen source, to achieve the maximum degradation of these azo dyes. While FT-IR, HPLC, and GC-MC analysis, as well as measuring the total organic carbon, were used to figure out how well the materials broke down. The novelty of this work was to uncover how the pure culture of *Streptomyces* strains breaks down these reactive azo dyes to fill in the gaps in our knowledge, leading to the development of eco-friendly ways to clean up the environment from the remaining azo dye residues.

## Materials and methods

### Chemicals and dyes

All chemicals used in this study for enrichment and discoloration assays were of analytical grade with desired purity (Hi-Media Lab., India; Sigma-Aldrich Ltd., USA). Different dyes used throughout the current study were kindly supplied by the Dye Star company, and their properties are summarized in Supplementary Table 1.

### *Streptomyces *isolation, screening, and cultural conditions

Soil samples were collected from different locations near textile dye-producing factories in Egypt. Samples were collected in a sterile polyethylene bag and stored in the fridge at 4 °C for further use. Samples were serially diluted according to Hayakawa and Nonomura [57], and 100 µL was plated on starch nitrate agar medium containing (g/L): Azo dye (RO122 or DB 15 or DB 38) 0.1, starch 10.0, K_2_HPO_4_ 1.0, MgSO_4_.7H_2_O 0.5, NaCl 0.5, KNO_3_ 2.0, CaCO_3_ 2.0, FeSO_4_.7H_2_O 0.01, agar 20.0, and were dissolved in 1 L distilled water. pH was adjusted to 7.0 [58]. Subsequently, the plates that had been inoculated were incubated at 28 ± 2 °C for 5 to 7 days. *Streptomyces* isolates were chosen based on the morphological characteristics of the colonies. The colonies surrounded by a clear zone were selected and inoculated in a liquid starch nitrate amended with different azo dyes to confirm their biodegradation ability [59]. The isolate that showed the most significant ability to degrade the three different azo dyes was selected for further testing.

### Decolorization assay

*Streptomyces* isolates were inoculated onto starch nitrate agar media supplemented with azo dye (0.2 g/L), specifically monoazo dye (RO 122), diazo dye (DB 15), and triazo dye (DB 38). The cultures were then incubated at 28 ± 2 °C for 5–7 days, and the dye decolorization was observed by the naked eye as a positive or negative result by forming a clear hallo zone around the growing colonies.

*Streptomyces* isolates that showed the best decolorization ability were inoculated on modified starch-nitrate broth media amended with 0.2 g/L of three different azo dyes. Then, 50 ml of media was inoculated with 1 mL of overnight actinobacterial culture and incubated under shaking at 140 rpm and 28 ± 2 °C for 7 days [60]. During incubation, aliquots of 3 mL culture medium were withdrawn after 3-, 5-, and 7days of incubation, then centrifuged at 5,000 rpm for 15 min to separate the cell mass, followed by filtration through a 0.45 μm syringe filter to remove any suspended particles. The decolorization of the dyes was assessed using a UV spectrophotometer by measuring the change in absorbance of the decolorization medium at 500 nm for reactive orange (122), 602 nm for direct blue (15) and 520 nm for direct black (38) against uninoculated control samples [61–63]. All decolorization experiments were done in three independent replicates.

The percentage of decolorization was calculated as follows:

$$\mathrm{Decolorization}\;(\%)=(\mathrm{initial}\;\mathrm{absorbance}-\mathrm{observed}\;\mathrm{absorbance})/\mathrm{Initial}\;\mathrm{absorbance}.\;\times\;100.$$

### Identification and characterization of *Streptomyces *isolate

The isolate that showed the highest decolorization ability was identified according to morphological, physiological, and biochemical features [64–67]. Also, molecular identification was performed by 16*S* rRNA gene sequencing. According to Coombs and Franco [68], the genomic DNA was extracted, and the 16 S rRNA gene was amplified using a polymerase chain reaction with the following primers: F: (5′ GAGTTTGATCCTGGCTCAG 3′) and R: (5′ GGTTACCTTGTTACGACTT 3′). The PCR reaction consists of 25 µL of 2× Taq PCR MasterMix, 2 µL of genomic DNA, and 1 µL of each primer (25 µM), and the final volume was completed to 50 µL with sterilized water. PCR amplification was performed with an initial denaturation step at 95 ºC for 4 min, followed by 35 cycles consisting of denaturing at 95 ºC for 30 s, annealing at 55 ºC for 30 s, and elongation at 72 ºC for 1 min; final extension at 72 ºC for 10 min; and ending at 4 ºC. The amplicons were resolved on a 1.5% agarose gel in 1x TBE buffer with 1 kb DNA ladder, purified, and sequenced using the same primer sets (Macrogen Co., Seoul, South Korea). The retrieved sequence was non-redundantly BLAST searched on NCBI database. The phylogenetic analysis was constructed with the neighbor-joining method by MEGA software portal [69], aligned with the Clustal muscle algorithm [70]. Selected sequences with the greatest similarity to the 16 S rRNA sequence of the *Streptomyces* isolate were aligned to generate the phylogenetic tree. The 16 S rRNA gene sequence of the isolate was deposited on GenBank databases with accession number #MW185782.

### Optimization of the most favorable parameters that affect the decolorization process

To achieve the highest decolorization rate of different azo dyes, such as monoazo dye (RO 122), diazo dye (DB 15), and triazo dye (DB 38), we optimized different parameters, dye concentration, incubation time, pH, and incubation temperature using *S. albidoflavus* 3MGH, following the previous work [28, 71–74] with slight modifications. We investigated each parameter separately, maintaining the others constant. .

### Optimization of dye concentration

The maximum decolorization of the three different azo dyes by the selected strain was tested at different concentrations (0.2, 0.3, and 0.4 g/L) in broth medium, and the percent of dye degradation was calculated as previously mentioned [28, 71, 72].

### Optimization of pH

Different pH levels were optimized to enhance the efficiency of dye decolorization by *S*. *albidoflavus* 3MGH using the broth culture method as previously described [28, 71–73]. We adjusted pH values (5, 6, 7, 8, and 9) in the medium using hydrochloric acid and sodium hydroxide.

### Optimization of incubation temperature

Different incubation temperatures (25, 30, 35, and 40 ˚C) were optimized to enhance dye degradation by the *S*. *albidoflavus* 3MGH in the broth culture as described previously [28, 71–74]. The effect of temperature on maximum dye degradation was investigated by calculating the percentage of dye decolorization by the test isolate at each temperature point, as previously mentioned [28, 72].

### Optimization of incubation time

The maximum decolorization of the three different azo dyes by the selected strain was tested at different incubation times (3, 5, 7, and 9 days) in broth medium and the percent of dye decolorization was calculated according to the previous studies [28, 71, 72].

### Impact of different carbon sources

To test the effect of different carbon sources (sucrose, glucose, mannose, xylose, maltose, fructose, arabinose, sorbitol, and galactose) on the rate of dye decolorization, 10 g/L of each sugar was used instead of starch in the original medium. Then, *S. albidoflavus* 3MGH was inoculated in the modified medium and incubated at 28 ± 2 °C for 5–7 days, and the percent of dye degradation was calculated according to Tripathi and Srivastava [72].

### Impact of different nitrogen sources

We tested the impact of different nitrogen sources, such as yeast extract, peptone, malt extract, and beef extract, on dye decolorization by replacing the original nitrogen source in the starch nitrate medium with 2 g/L from each source, followed by medium inoculation and a dye decolorization assay, as previously described by Kameche et al. [75].

### Determination of extracellular laccase

Laccase enzyme was screened on an extracellular extract of *S. albidoflavus* 3MGH. First, the strain was grown in starch nitrate broth medium for 5 days at 28 ± 2 °C, followed by centrifugation at 5,000 rpm for 10 min at 4 ℃ to separate the cell mass. The resultant supernatant was filtered through a 0.45 μm syringe filter to remove any suspended particles and used as a crude laccase enzyme [76, 77]. Then, laccase activity was assayed as shown earlier by [78, 79] depending on the oxidation of ABTS (2, 2-azinobis-(3-ethylbenzthiazoline-6-sulphoate) with slight modification. The reaction mixture consisted of 200 µL of ABTS (0.5 mM), 200 µL of crude enzyme, and 2400 µL of sodium acetate buffer (pH 4.5, 100 mM). The reaction was incubated at 25 ℃ for 10 min and stopped by 10% TCA. Then the blue color resulting from the oxidation of ABTS by the laccase enzyme was measured at 420 nm. One unit of laccase was defined as the amount of enzyme capable of oxidizing 1 µM of substrate (ABTS) per minute. Protein concentration was assayed by the Bradford method [80] using bovine serum albumin as an authentic standard.

### Analysis of metabolites after dyes decolorization

Total organic carbon (TOC) and its reduction ratio were used to evaluate dye mineralization. After centrifugation and filtration to remove cell biomass, the presence of total organic carbon (TOC) in dye-containing culture broths was determined using standard method No. 5310. TOC was calculated under optimum incubation conditions using the standard method. The following equation was used to calculate the removal ratio.

$$TOC\;Removal\;Ratio\;\left(\%\right)=\frac{{Initial\;TOC}_{\left(0h\right)}-{Observed\;TOC}_{\left(t\right)}}{{Initial\;TOC}_{\;\left(0h\right)}}\times\;100$$

The TOC (0 h) and TOC (t) represent the initial TOC values at 0 times and after a specific incubation time (t), respectively. High-Performance Liquid Chromatography (HPLC) analysis of control dyes and their decolorized metabolites was done with an HPLC engine fully equipped with a Waters™ 2690 (Waters Limited, Hertfordshire, UK) equipped with a C18 column (symmetry, 4.6 × 250 mm) with a mobile phase consisting of ethanol (55%), deionized water (45%), a flow rate of 0.80 mL/min for 10 min, and a UV detector set at 280 nm modifying the procedure reported earlier [7]. A total of 10 µL of mono (RO 122), di (DB 15), and tri (DB 38) azo dyes and their degradation metabolites were injected into the column, and the chromatogram profile was observed. A Fourier transform infrared (FT-IR) analysis was performed for the controls and extracted by-product samples after they were freeze-dried and mixed with KBr pellets using the FT-IR Spectrum 2000 Perkin-Elmer spectrometer. The spectra were collected within a scanning range of 400–4000 cm^-1^ [81]. Gas chromatography-mass spectrometry (GC-MS) analysis was done by QP2010 gas chromatography combined with mass spectroscopy (an Agilent 6890 Series) by modifying the procedure reported earlier [82]. The experiment was conducted using a Rested column measuring 0.25 mm in diameter and 30 m in length, with an ionization voltage of 70 eV. The temperature of the column was initially adjusted to 40 °C and maintained for a duration of 4 min. Subsequently, the temperature was increased linearly at a rate of 10 °C per minute until it reached 270 °C. Once the desired temperature was attained, it was maintained for an additional 4 min. The temperature of the injection port was maintained at 275 °C, while the mass interface was maintained at 300 °C. A carrier gas consisting of helium was employed at a flow rate of 1 mL/min for a duration of 30 min.

### Deposition of the *Streptomyces *isolate

The sequence of the most potent azo dye-degrading *Streptomyces* isolate, *Streptomyces albidoflavus* 3MGH, was deposited into Genbank with accession #MW185782.1.

### Statistical analysis

The data were analyzed using one-way ANOVA in GraphPad Prism version 5, and the results were expressed as the mean of three replicates ± standard deviation.

## Results

### Isolation, screening, and identification of *Streptomyces*

**Fig. 1:**
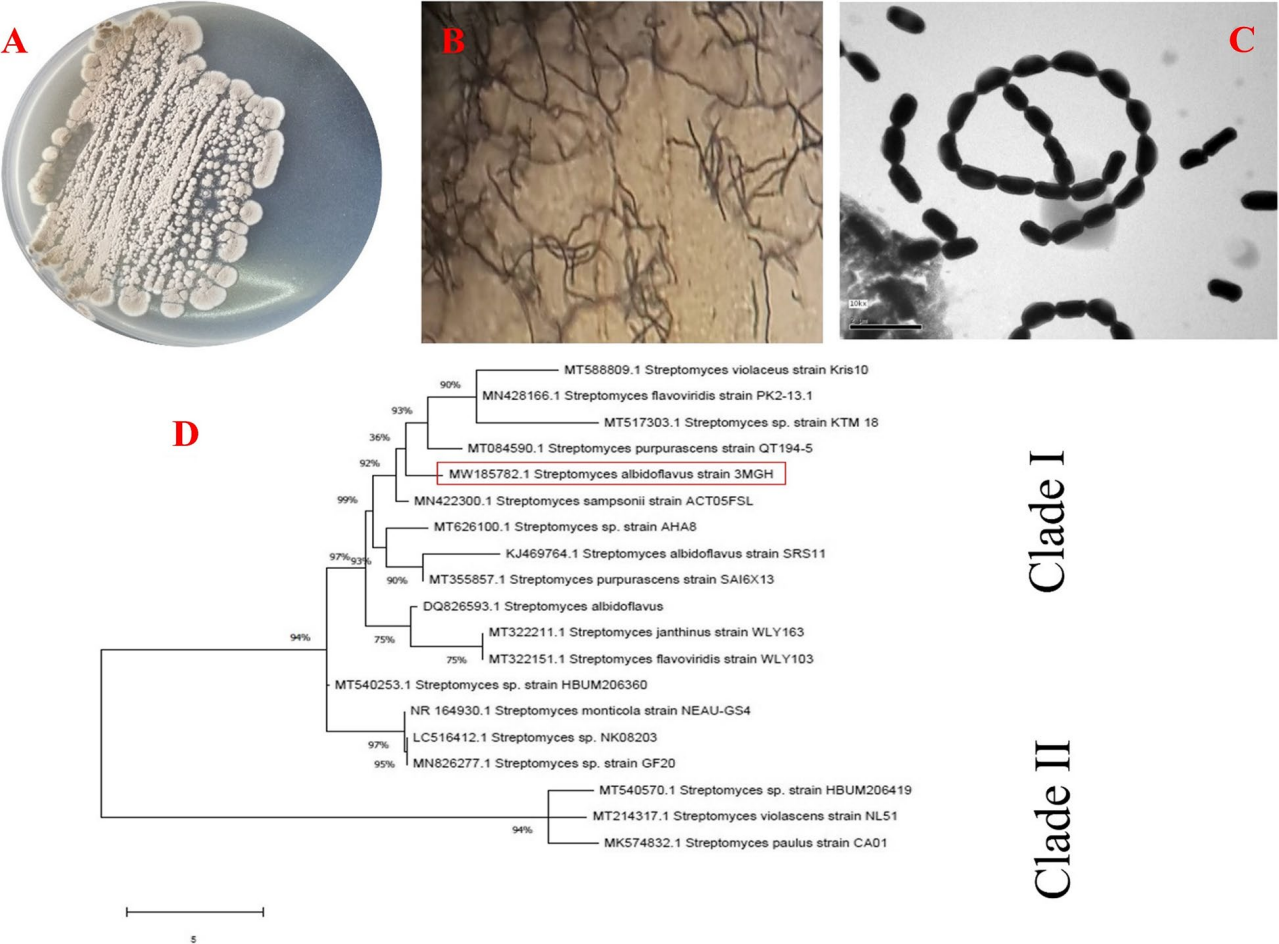
Morphological and molecular identification of isolate A7 showing cultural view (**A**), flexuous sporephores hyphae (**B**), and Transmission electron microscope (TEM) photomicrograph showing smooth spore surface (**C**). Molecular phylogenetic analyses of *S. albidoflavus *3MGH (**D**) by Maximum Likelihood Model of MEGA 7.0 package

 Twenty-five Streptomyces isolates were isolated and given the names A 1 to A 25 due to their distinct morphological and cultural characteristics (data not shown). Among these strains, A7 showed higher decolorization potency compared to other isolates when tested on RO122, DB 15, and DB 38 dyes. Based on its morphological and cultural characteristics (Table 1), A7 was identified as *Streptomyces* sp. 3MGH and selected for molecular identification through DNA sequencing. Morphologically, *Streptomyces* sp. 3MGH showed straight, flexuous spore chain morphology with smooth spore surface ornamentation (Fig. 1A-C). The isolate showed no ability for diffusible or melanoid pigment production, with different abilities in carbon sources utilization (Table 1).

**Table 1:**
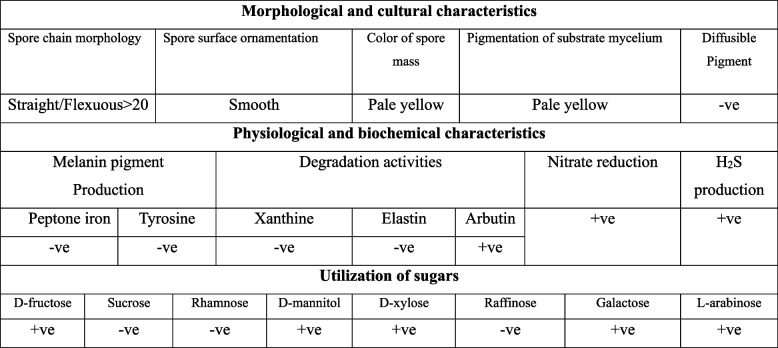
Morphological, physiological, and biochemical characteristics of isolate A7

### Molecular identification of selected *Streptomyces *isolates

The identification of *Streptomyces* sp. 3MGH was confirmed through molecular analysis of the 16 S rRNA partial sequence using its genomic DNA as a template for PCR, as outlined in the Materials and Methods section. The multiple sequence alignment analysis revealed that this *Streptomyces* isolate showed a high level of similarity with previously identified *Streptomyces albidoflavus* strains available in the NCBI GenBank database (https://www.ncbi.nlm.nih.gov/genbank/). As a result, the isolate was deposited in the database with accession number MW185782.1. Furthermore, a molecular phylogenetic analysis was conducted using the MEGA 7.0 software package, including the deposited isolates from the database, to establish the phylogenetic tree (Fig. 1D).

### Effect of different parameters on decolorization of different functional azo dyes by *Streptomyces albidoflavus *3MGH

Abiotic factors such as temperature, pH, salinity, nutrient availability, and initial dye concentration significantly influence the decolorization of dyes. In this study, we assessed the impact of these abiotic factors on the dye decolorization potential of *S. albidoflavus* 3MGH.

### Effect of different incubation temperature

**Fig. 2:**
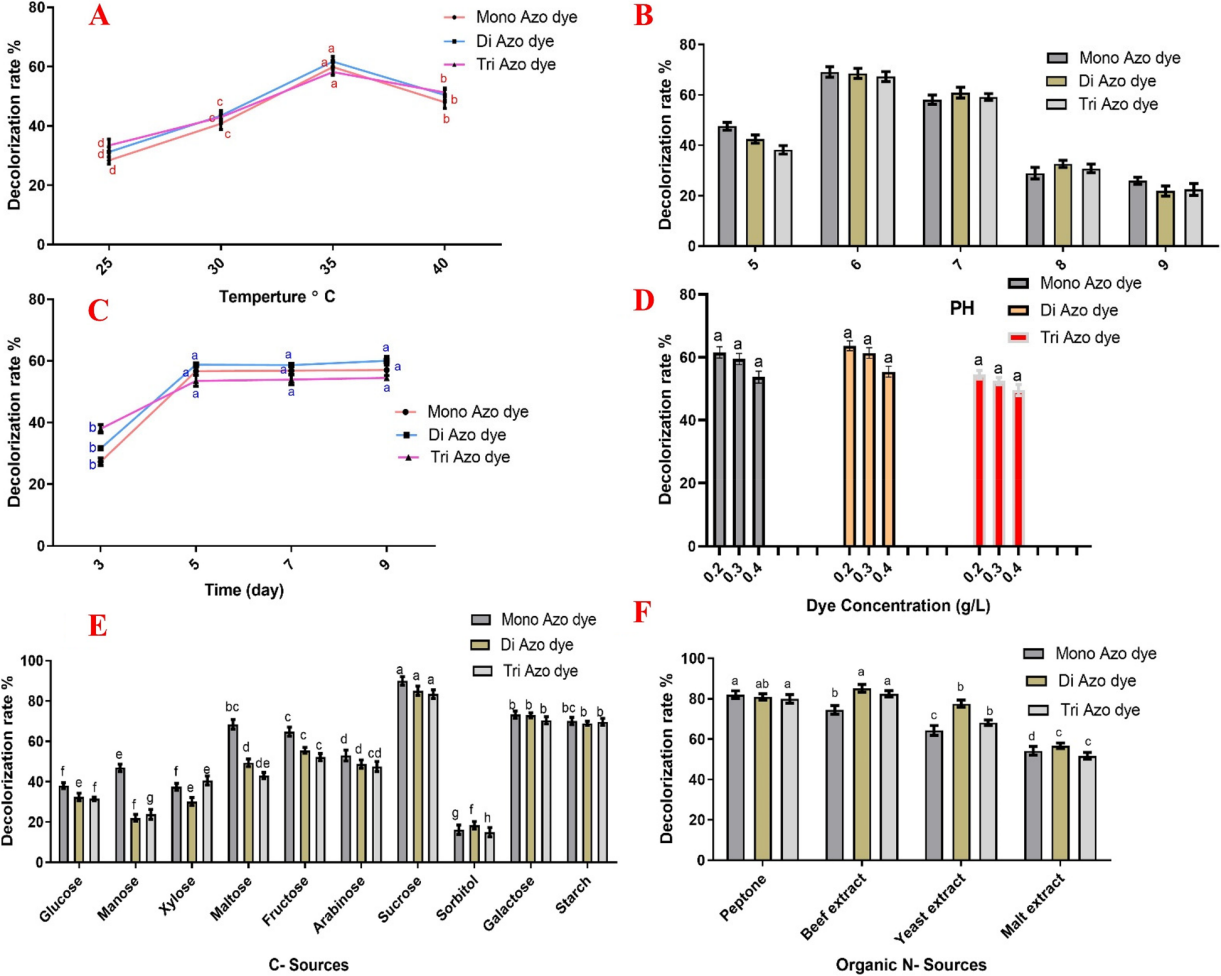
Effect of different incubation temperatures (**A**), pH of the culture medium (**B**), Incubation time (**C**), Dye concentration (**D**) different carbon sources (**E**), different nitrogen sources (**F**) on RO 122, DB 15, and DB 38 azo dyes by *S. albidoflavus* 3MGH. different letters are significantly different at *P* < 0.05 according to Tukey’s test

Figure 2A shows the decolorization ability of S. albidoflavus 3MGH at different incubation temperatures. Increasing the temperature from 25 °C to 35 °C had a significant effect on azo dye biodegradation. The optimum temperature was 35 °C, with decolorization rates of 59.87%, 61.71%, and 58.2% for RO 122, DB 15, and DB 35 azo dyes, respectively. While 40 °C resulted in decreased activity with 48.01%, 50.43%, and 51.29% for RO 122, DB 15, and DB 35 azo dyes, respectively.

### Effect of different pH

The effect of pH on the decolorization rate of the three azo dyes by S. albidoflavus 3MGH was studied in the pH range 5.0 to 9.0 and as shown in Fig. 2B, the maximum depolarization rate was achieved at pH 6, with decolorization rate of 69.1%, 68.52%, and 67.3% for RO 122, DB 15, and DB 35 azo dyes, respectively. However, the decolorization rate dropped sharply as pH increased from 7 to 9, which is significantly lower than biodegradation in the case of pH 6.

### Effect of different incubation time

The decolorization rate of the three different azo dyes was studied at different incubation times (3, 5, 7, and 9 days). The results showed that on day five, the decolorization rate was 56.62%, 58.75%, and 53.49, which was significantly higher than those after 3 days (27.23%, 31.57%, and 37.95% for RO 122, DB 15, and DB 35 azo dyes, respectively). Compared to day 5, there was no significant increase after 7 and 9 days (Fig. 2C).

### Effect of different dye concentrations

The decolorization ability of S. albidoflavus 3MGH was examined at different concentrations of azo dye (0.2, 0.3, and 0.4 g/L). After the incubation period, the maximum decolorization rate was achieved at a concentration of 0.2 g/L with 61.56%, 63.7%, and 54.48% for RO 122, DB 15, and DB 35 azo dyes, respectively. However, as the dye’s initial concentration increased (0.3–0.4 g/L), the decolorization rate gradually declined (Fig. 2D).

### Effect of different carbon sources

Any additional carbon or nitrogen sources can largely affect the bio-decolorization ability of microorganisms. So, virous carbon sources such as glucose, mannose, xylose, maltose, fructose, arabinose, sucrose, sorbitol, galactose, and starch) were used to study their effect on the decolorization rate of the three azo dyes by S. albidoflavus 3MGH. After the incubation period, results showed that sucrose was the best C- source, increasing azo dye decolorization significantly more than other sources, giving 89.79%, 85.06%, and 83.44% for RO 122, DB 15, and DB 35 azo dyes, respectively (Fig. 2E).

### Effect of different nitrogen sources

The effect of different nitrogen sources (peptone, beef extract, yeast extract, and malt extract) was examined, and the obtained results showed that peptone and the beef extract were the best organic N- sources for significantly increasing azo dye decolorization (82.04%, 80.91%, and 79.9%), (74.51%, 85.12%, and 82.48%), respectively, for RO 122, DB 15, and DB 35 azo dyes (Fig. 2F).

### Determination of laccase activity

Various enzymes, including laccases, control the bio-decolorization of Azo dyes. So, the extracellular laccase of *S. albidoflavus* 3MGH was assessed, and the results showed that total laccase activity was 21.13 U, while the total protein was 3.54 mg, giving a specific activity of 5.96 U/mg.

### Evaluation of the decolorized metabolite products of three incubated azo dyes with *S. albidoflavus *3MGH

#### Total organic carbon

The results of the TOC analysis of the decolorized products of the three tested dyes before and after incubation with *S. albidoflavus* 3MGH at optimum conditions are shown in Table 2. Mono-azo dye (RO 122) was found to have the highest value for organic carbon reduction, indicating promising results. The removal ratio was reduced to a lower level using di (DB 15) and tri (DB 38) azo dyes.

**Table 2:** TOC and TOC Elimination Ratio (%) of azo Dyes before and after treatment with *S. albidoflavus* 3MGH under optimum condition

Sample	TOC (ppm)	TOC Elimination Ratio (%)
Control Mono Azo Dye (Reactive Orange 122)	336	94.44
Incubated Mono Azo Dye (Reactive Orange 122)	18.67	
Control Di Azo Dye (Direct Blue 15)	541.33	86.26
Incubated Di Azo Dye (Direct Blue 15)	74.67	
Control Tri Azo Dye (Direct Black 38)	410.67	68.18
Incubated Tri Azo Dye (Direct Black 38)	130.67	

#### High-performance liquid chromatography (HPLC) analysis

Supplementary Fig. 1A-F illustrate the HPLC chromatogram profiles of monoazo dye (RO 122), diazo dye (DB 15), and triazo dye (DB 38) before and after treatment with *S. albidoflavus* 3MGH under optimum conditions. The chromatogram of the monoazo dye (RO 122) before incubation (control) with *S. albidoflavus* 3MGH at optimum conditions revealed two major peaks at retention times of 4.40 and 6.80 min, compared to the complete disappearance of these peaks and the appearance of new minor peaks at retention times of 3.8, 6.91, and 6.4 in the dye chromatogram after incubation (treated). Supplementary Fig. (1 A&B) Furthermore, peaks disappear in the HPLC di Azo dye (DB 15) chromatogram at retention times of 4.04, 5.6, 6.3, 8.2, 9.1, 9.4, 9, and 10.9 min before incubation (control), whereas other peaks appear at 3.8 (minor) and 6.9 min (major) after incubation (treated). Supplementary Fig. (1 C&D). The triazo dye (DB 38) exhibits similar behavior. The disappearance of all peaks observed in the chromatogram before incubation (control) at retention times 1 (minor), 1.4 (minor), 4.4 (major), 5.2 (minor), 5.7 (minor), 7.7 (major), 12.3 (minor), and 15.4 min (minor), respectively, and the formation of two peaks in the chromatogram after (treated) incubation with *S. albidoflavus* 3MGH at times 7.2 (minor) and 7.6 min (major), respectively (Supplemental Fig. 1E&F).

#### FT-IR spectra analysis

**Fig. 3:**
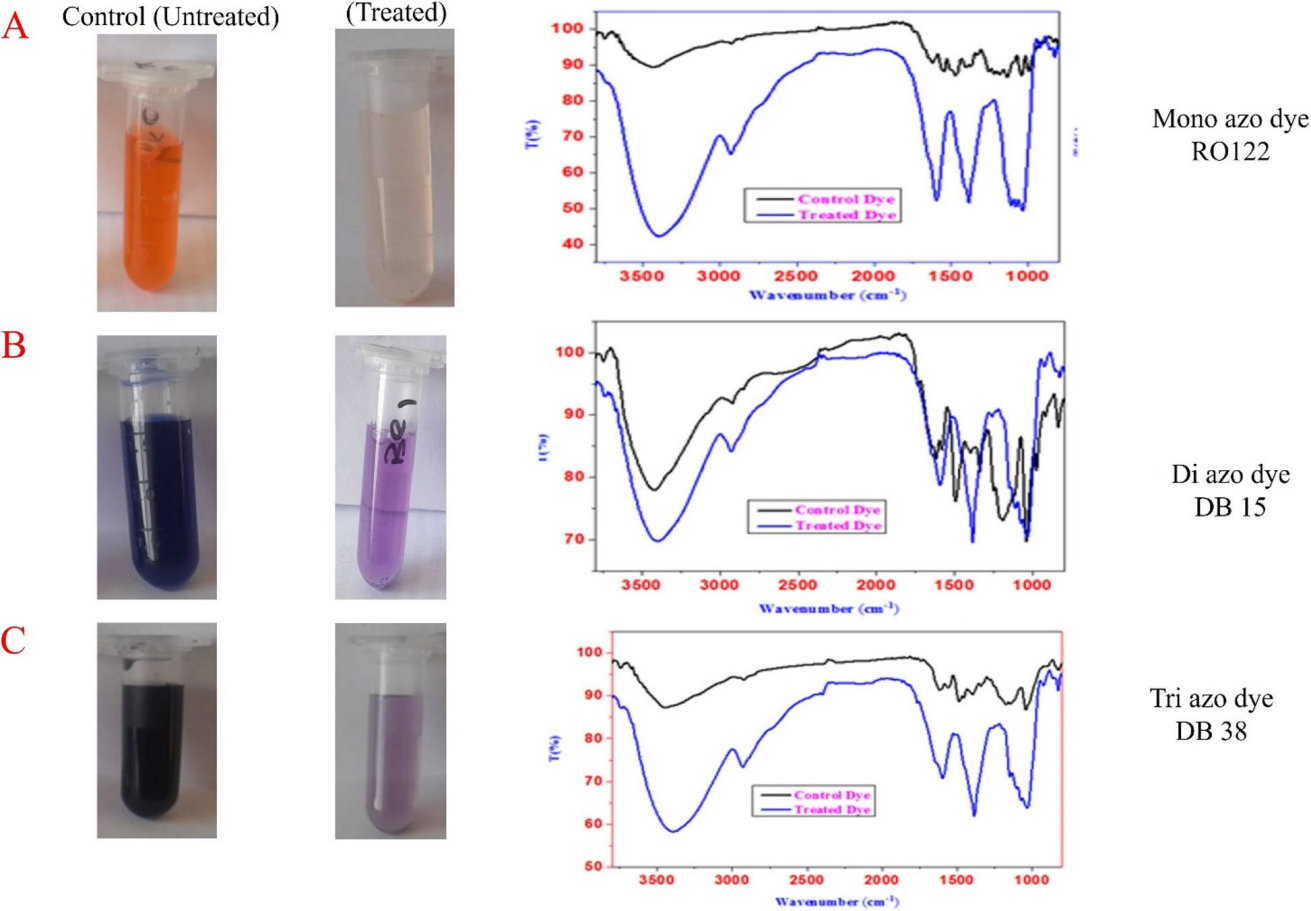
FT-IR spectra and photographic images of **A** mono azo dye (Reactive Orange 122), **B** di azo dye (Direct Blue 15) and **C** tri azo dye (Direct Black 38) before and after incubation with *S. albidoflavus* 3MGH at optimum conditions

Figure 3A-C show the FT-IR spectra at optimal conditions for control monoazo (RO 122), diazo (DB 15), and triazo (DB 38) dyes and their degraded metabolite products. Significant changes in the IR spectrum of treated dyes include the elimination, addition, and shift of peaks caused by *S. albidoflavus* 3MGH decolorization. Furthermore, the new peaks at frequencies lower than 1600 cm^-1^ could be the result of azo dye degradation byproducts and the introduction of new functionalities [81]. After incubation with *S. albidoflavus* 3MGH, the 18 peaks of the (RO 122) FT-IR chart were reduced to about 7 peaks (Fig. 3A). Similarly, the di-azo dye (DB 15) peak was reduced from 12 to near 7 (Fig. 3B). Finally, within the sample incubated with *S. albidoflavus* 3MGH, the 12 peaks on the FT-IR chart that corresponded to the control triazo dye (DB 38) were reduced to nearly 7 peaks (Fig. 3C).

The FT-IR spectra of the control dye RO122 (Fig. 3A) revealed specific absorbance bands at wavelengths: 3422 cm^−1^ for stretching -NH_2_, 2938 cm^−1^ for stretching alkanes, 2349 cm^−1^ for Si-H silane and/or NH + vibrations of charged amine derivatives, 1608 cm^−1^ for C = C, 1561 cm^−1^ for-N = N-, 1475 cm^−1^ for -C-H &C = S stretching, 1390 cm^−1^ for -O-H and C-N vibrations in aromatic amines, 1200 cm^−1^ for SO_3_, 1143 cm^−1^ for S = O, 753 cm^−1^ for S-OR, and 620 cm^−1^ for C-Cl. The FTIR spectra of metabolites gathered after decolorization of dye RO122 (Fig. 3A) revealed absorbance bands at wavelengths of 3403 cm^−1^ for stretching -NH_2_, 3014 –2871 cm^−1^ for C-H, =C–H (benzene ring), 1618 cm^−1^ and 1490 cm^−1^ for stretching C–C, and 1054 cm^−1^ for stretching C-O.

The FT-IR spectrum of the control dye DB 15 (Fig. 3B) also showed specific absorbance bands at wavelengths of 3403 cm^−1^ for stretching -NH_2_, 2957 cm^−1^ for stretching C-H, 1608 1570 cm^−1^ for C = O, 1599 cm^−1^ for N = N-, 1399 cm^−1^ for stretching -C-H and C = S, 1114 cm^−1^ for stretching S = O, and 829 cm^−1^ for S-OR. While the FTIR spectra of metabolites, obtained after decolorization of dye DB 15 (Fig. 3B) revealed absorbance bands at wavelengths of 3412 cm^−1^ for stretching -NH_2_, 2938 cm^−1^ for C-H, =C–H (benzene ring), 1646 cm^−1^ for stretching C–C or C = O, 1371 and1133 cm^−1^ for stretching C–N, and 1019 cm^−1^ for stretching C-O.

Moreover, the FT-IR spectrum of the control dye DB 38 (Fig. 3C) showed specific absorbance bands at wavelengths of 3450 cm^−1^ for stretching -NH_2_, 1608 cm^−1^ for C = C, 1570 cm^−1^ for-N = N-, 1494 cm^−1^ for N = O, 1342 cm^−1^ for -O-H and C-N vibrations in aromatic amines, 1181 cm^−1^ for SO3^*−*2^, 1048 cm^−1^ for S = O, and 829 cm^−1^ for S-OR. While the FT-IR spectra of metabolites assembled after decolorization of dye DB 38 (Fig. 3C) demonstrated absorbance bands at wavelengths of 3403 cm^−1^ for stretching -NH_2_, 2938 cm^−1^ for C-H, =C–H (benzene ring), 1637 cm^−1^ for stretching C–C, 1390 and 1114 cm^−1^ for stretching C–N, and 1029 cm^−1^ for stretching C-O.

#### GC-MS analysis

The GC-MS technique was used to provide more information about the metabolic pathways of the tested dyes during decolorization under the action of *S. albidoflavus* 3MGH. The interpretation of metabolic intermediate products may help clarify signs that may explain the possible biodegradation mechanism. Gas chromatograms of decolorized dye metabolites revealed a large number of these peaks, but some peaks were identified using a mass spectrum. However, the majority of the fragmentation patterns of the tested decolorized dye products have a low molecular weight (M/Z values). This could be referring to the efficiency of the mineralization process.

**Fig. 4:**
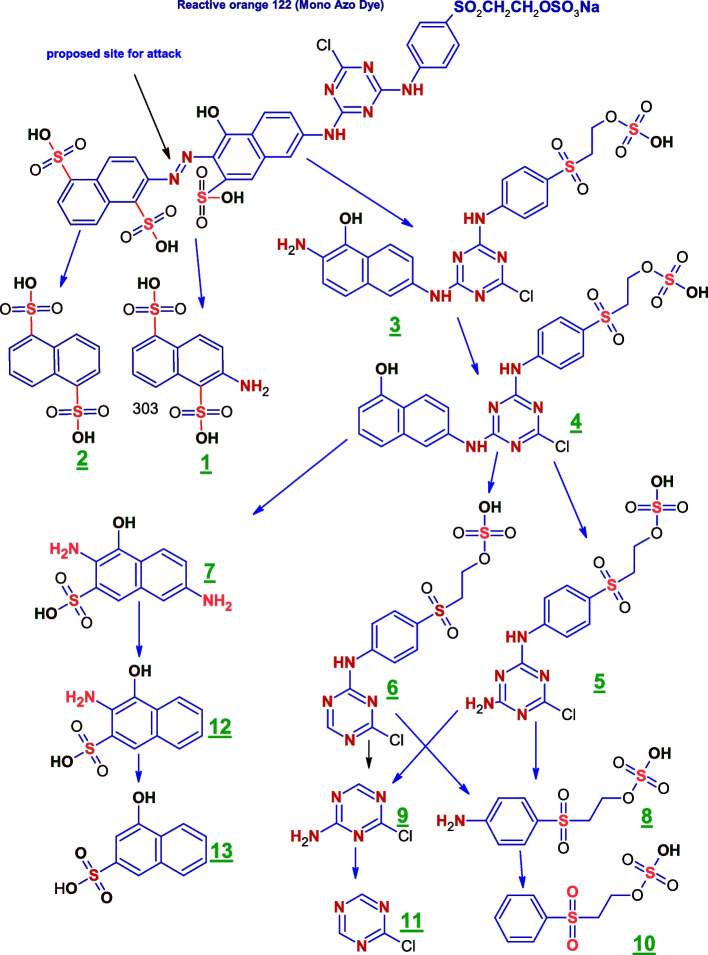
Scheme for the proposed mechanism for degradation process of mono azo dye (reactive orange 122) through reductive cleavage of azo bonds by the action of *S. albidoflavus* 3MGH under optimum condition

**Fig. 5:**
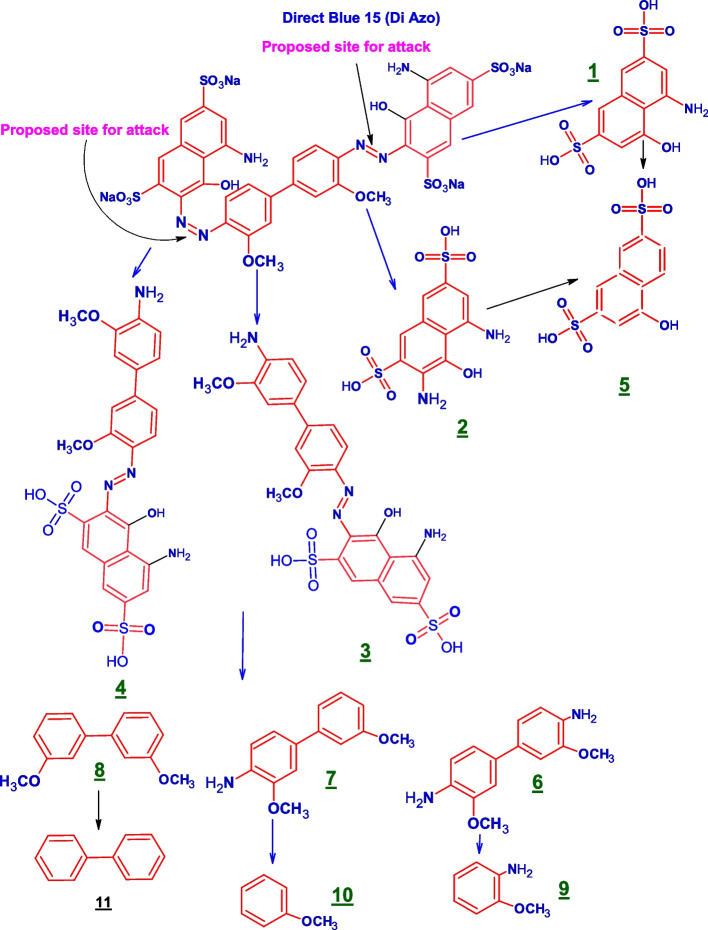
Scheme for the proposed mechanism for degradation process of di azo dye (DB 15) through reductive cleavage of azo bonds by the action of *S. albidoflavus* 3MGH under optimum condition

**Fig. 6:**
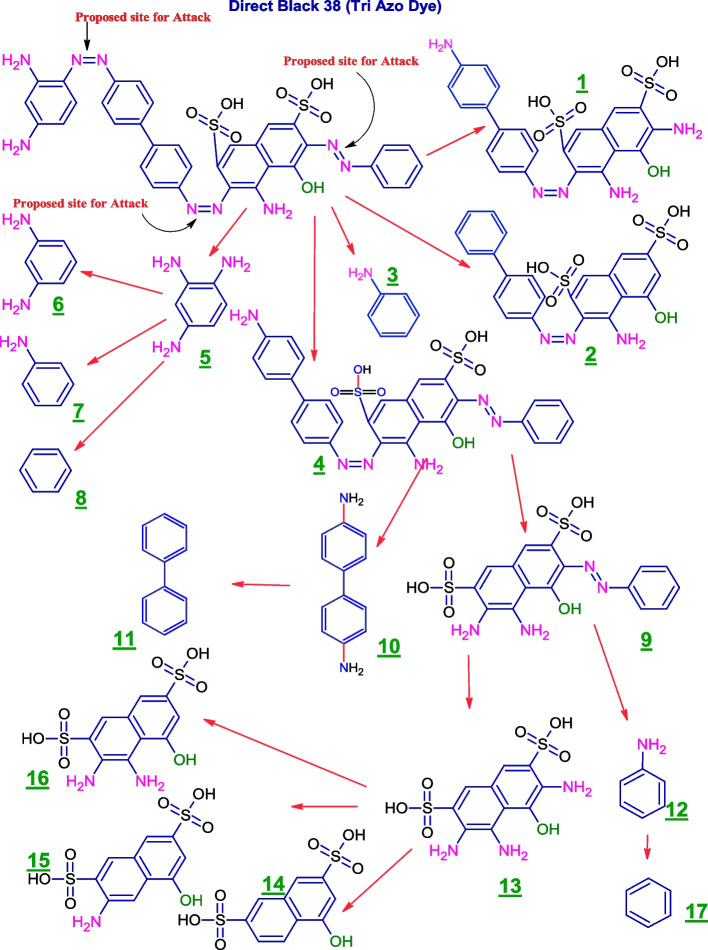
Scheme for the proposed mechanism for degradation process of tri azo dye (DB 38) through reductive cleavage of azo bonds by the action of *S. albidoflavus* 3MGH under optimum condition

 Reductive cleavage of the azo linkages may break down the tested dyes monoazo (RO 122), diazo (DB 15), and triazo (DB 38), resulting in different intermediate fragmentation products, as shown in Figs. 4, 5 and 6. Furthermore, GC-MC mass charts detected the decolorized metabolite fragmentations of reactive orange 122, direct blue 15, and direct black 38, and their retention times (RT) were reported in Tables 3, 4 and 5, respectively.

**Table 3:**
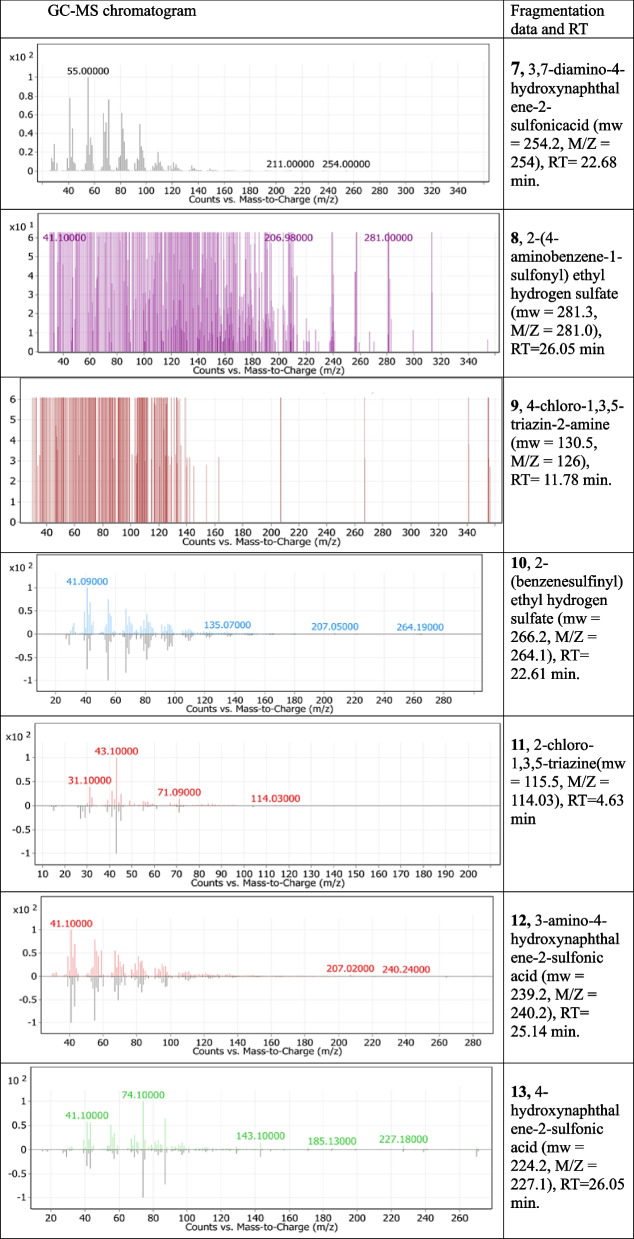
GC-MS chromatograms of the intermediate metabolite products and their corresponding chemical data and retention times (RT) of monoazo dye (RO 122) after treatment with *S. albidoflavus* 3MGH at optimum decolorization conditions

**Table 4:**
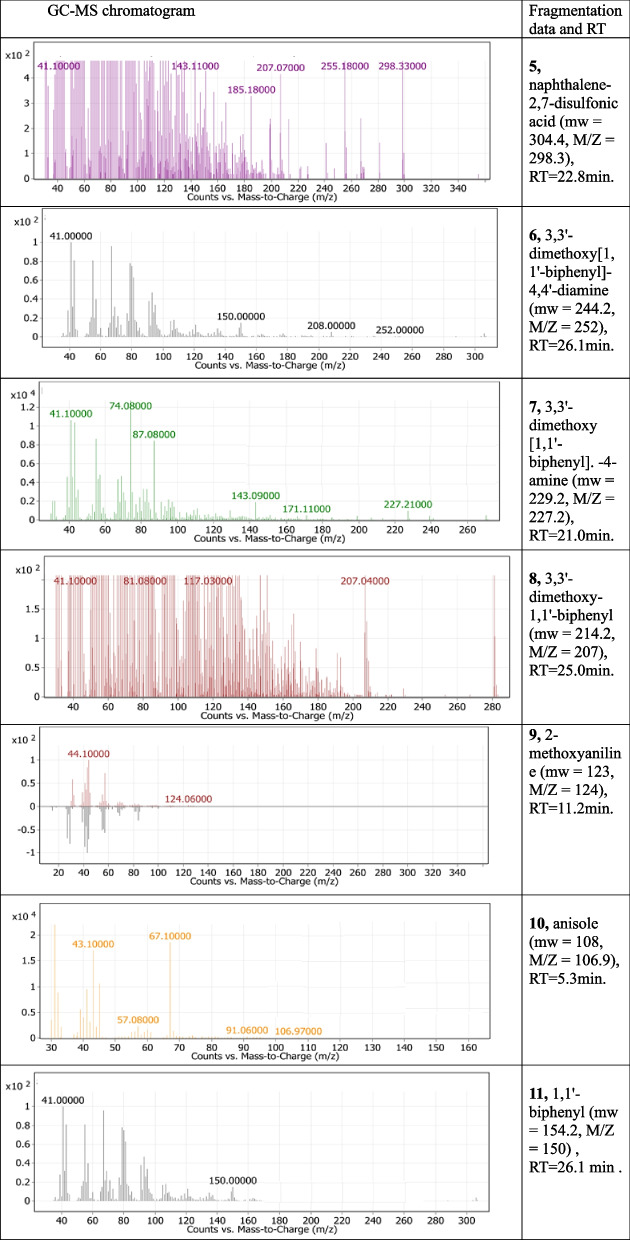
GC-MS chromatograms of metabolites intermediate products and their corresponding chemical data and retention time (RT) of di azo dye (DB 15) after treatment with *S. albidoflavus* 3MGH under optimum decolorization conditions

**Table 5:**
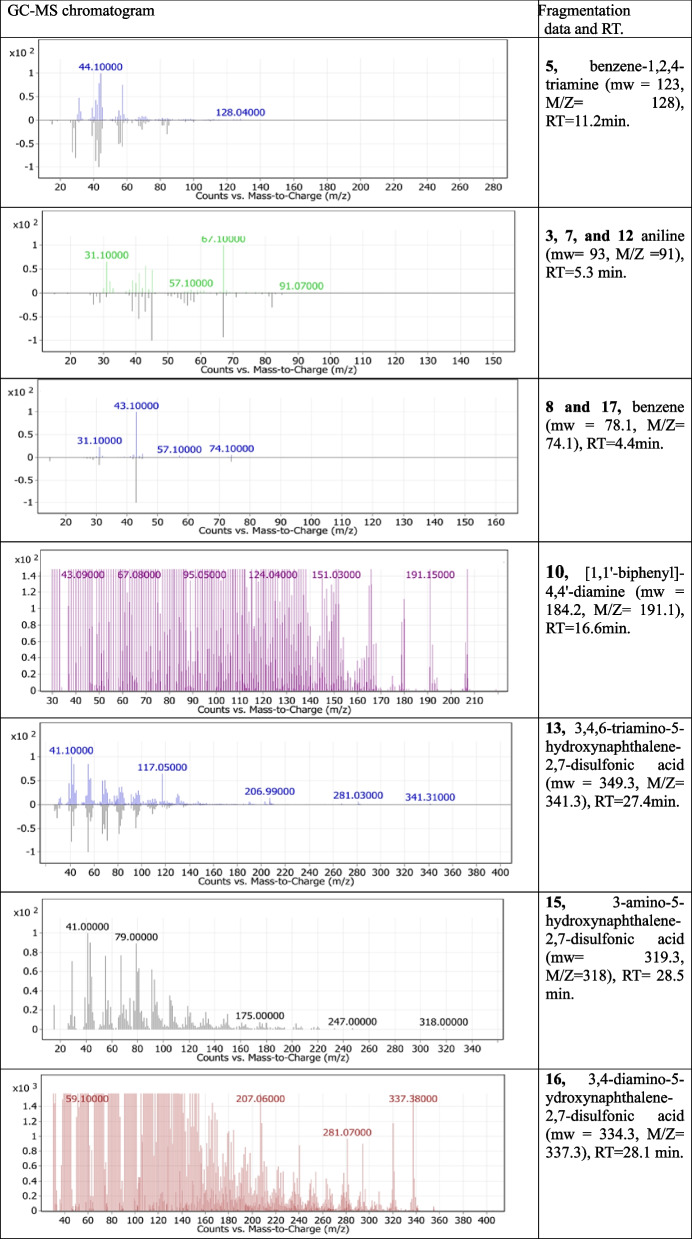
GC-MS chromatograms of metabolites intermediate products and their corresponding chemical data and retention time (RT) of tri azo dye (DB 38) after treatment with *S. albidoflavus* 3MGH under optimum decolorization conditions

## Discussion

Azo dyes are widely employed in various industries, such as textiles and cosmetics, posing a significant concern due to their recalcitrant nature and that of their byproducts. The indiscriminate release of untreated effluents containing these dyes into the environment has emerged as a critical problem [83]. Approximately 3 × 10^5^ tons of discharged textile dyes are added to the global water environment [84]. Thus, the aim of the current study was the isolation and screening of *Streptomyces* spp. capable of decolorizing different types of Azo dyes. Among twenty-five isolates of *Streptomyces* from different sources of soil polluted with textile effluents in Egypt, isolate A7 showed the highest decolorization activity. Based on its morphological and biochemical characteristics (Table 1), it was identified as *Streptomyces* sp. 3MGH. The morphological identity of *Streptomyces* sp. 3MGH was confirmed from the 16 S rRNA gene fragment. The PCR amplicon of the 16 S rRNA region was sequenced and showed a higher similarity with the corresponding database-deposited isolates of *Streptomyces albidoflavus*. The 16 S rRNA sequence of *S. albidoflavus* 3MGH was deposited into the GenBank with accession MW185782.1 Fig. 1A-D. Recent studies have revealed that actinobacteria, especially *Streptomyces* species, exhibit a remarkable ability to decolorize azo dyes [53, 54, 85]. The bio-decolorization ability of other actinobacterial species was reported by *Nocardia* sp. against Congo red dye [86].

Different abiotic factors, including temperature, pH, incubation time, nutrient availability, and initial dye concentration, can affect the decolorization rate [39]. In this study, we assessed the impact of all these abiotic factors on the dye decolorization ability of *S. albidoflavus* 3MGH. Temperature plays a crucial role in the optimal ability of microorganisms, as it affects both their growth and enzymatic activity, which, in turn, may influence their decolorization ability. Figure 2A shows that *S. albidoflavus* 3MGH achieved the best decolorization rate at 35 °C for all tested dyes (RO122, DB 15, and DB 38). Previous studies highlighted the effect of temperature on decolorization rate and how temperature can affect the microbe’s physiology [85, 87–90]. Our results are in harmony with those of Khan and Malik, who also observed an increased bio-decolorization rate of RB5 dye in the mesophilic temperature range [91, 92]. Endo (2003) reported that at 40 °C, *Streptomyces* sp. can achieve its best decolorization rate. In the same line, *Nocardia alba* demonstrated the greatest decolorization ability for Reactive Orange 16 at 30 °C [93]. While Kameche et al. (2022) showed that four *Streptomyces* strains produced the highest decolorization ability for diazo dye Evans blue at a temperature range of 30–40 °C.

During the process of bio-decolorization, the pH of the medium significantly affects the transport of dye molecules within bacterial cells. The presence of H^+^ ions in the medium can inhibit the dye cations, thereby reducing the efficiency of the dye decolorization process [5, 94]. We studied the effect of pH on the decolorization rate of the three azo dyes by the selected isolate, and found that pH 6.0 achieved the maximum depolarization rate (Fig. 2B). Our study’s results are consistent with previous research findings [89, 92, 95].

Studies have indicated that actinobacterial strains often exhibit their optimum degradation activity at neutral pH values or slightly alkaline pH values [96]. Conversely, at strongly acidic or strongly alkaline pH values, the rate of discoloration decreases rapidly due to chemical changes in the azo dye’s structure caused by protonation or deprotonation of azo bonds [97]. The incubation time required for an effective decolorization process is a crucial factor, and it varies depending on the tested strains [98]. Additionally, Siddeeg et al. [99] found that as the incubation period increases, the decolorization becomes more pronounced, leading to a higher bacterial biomass. The current results demonstrated the achievement of the maximum decolorization rate on day five. While there was no significant increase after 7 and 9 days compared to day 5 (Fig. 2C).

The decolorization rate of the three azo dyes (RO122, DB 15, and DB 38) was studied at different dye concentrations (0.2, 0.3, and 0.4 g/L), and the maximum decolorization rate was achieved at a concentration of 0.2 g/L for the tested azo dyes (Fig. 2D). The findings suggest that the decolorization process is significantly affected by the initial dye concentration. As the dye concentration increases, the rate of decolorization decreases. This could be attributed to the natural toxicity of higher dye concentrations, which can inhibit microbial activity in most organisms [96, 100]. Our results are in harmony with previous studies that showed a decreased decolorization rate with increasing dye concentration [39, 72, 75]. Also, previous studies have demonstrated the decolorization capabilities of certain microorganisms isolated from dying wastewater with a specific dye concentration. At a concentration of 250 mg/L, *Nocardiopsis alba* demonstrated an 85% decolorization rate of reactive orange 16 [93]. In another study, *Streptomyces* sp. VITDDK3 effectively degraded azo dye reactive red 5B at a concentration of 50 mg/L [101]. These results highlight the potential of these microorganisms for dye decolorization in wastewater treatment applications.

The efficiency of microbial bio-decolorization is significantly influenced by the availability of supplementary carbon and nitrogen sources. These additional nutrients play a crucial role in providing energy to the cells, supporting their growth, survival, and metabolic processes [95, 102]. After the incubation period, our results showed that sucrose was the best C-source for increasing azo dye decolorization significantly compared to other sources **(**Fig. 2E), and for N-sources, peptone and beef extract were the best organic N-sources for significantly increasing azo dye decolorization (Fig. 2F) for all tested azo dyes. Previous work by Kameche et al. [75] showed that glucose, followed by sucrose, were the best C-sources, and ammonium chloride and yeast extract were the best nitrogen sources for bio-decolorization of RB5 within 72 h by *Priestia* sp. RA1. While, Vinayak and Singh [39] showed that the best carbon source was glucose, followed by sucrose and fructose, in the bio-decolorization of Evans blue azo dye using four different Streptomyces spp. In contrast, Lakshmaiah et al. [92] reported lactose to be the best carbon source and yeast extract to be the best nitrogen source for the bio-decolorization of reactive blue 222 azo dye by *Kucoria marina* CU2005.

Laccases have been extensively researched for their ability to degrade azo dyes. They are classified as multi-copper phenol oxidases and decolorize azo dyes using a highly nonspecific free radical mechanism. This process results in the formation of phenolic compounds while avoiding the production of toxic aromatic amines [103]. In this study, isolate A7 was subjected to quantitative screening for laccase production, and the results indicated a specific activity of 5.96 U/mg. Actinomycetes, particularly *Streptomyces* sp., are known to be proficient laccase producers. Previous research has focused on the purification and characterization of laccases from various Actinomycetes, including *Streptomyces* species such as *S. cyaneus* and *S. lavendulae* [104, 105]. Studies have indicated that the process of decolorization can be affected by the redox potential of microbial enzymes and their activities on the active sites of pollutants [106].

Considering the obtained results during the optimization of different parameters affecting the bio-decolorization process of the three dyes, the diazo dye either slightly improved or had comparable results to the monoazo dye, while the triazo dye always came in third position. This is because of how the chemical structures of the dyes affect the decolorization process. As a result, dye solutions’ decolorization rates varied, primarily due to the proportion of azo linkages. The chemical structure of the dye, particularly the azo bond number, functional groups, benzene ring locations, and morphological nature, has been directly associated with variations in decolorization rates. Low-molecular-weight, simple azo dye structures frequently decolorize faster than high-molecular-weight, complicated dye structures [107]. Therefore, despite having a significantly lower molecular weight than RO 122 and direct blue 15, direct black 38 had a slower rate of color evaporation. This could be due to the fact that it carries three azo bonds, which complicate its structure and hinder decolorization.

However, the difference between them is not significant in general. This could imply that *S. albidoflavus* 3MGH may be utilized for the treatment of a wide range of dyes and colored effluents from industrial facilities. By observing the results of the total organic carbon removal of the three tested dyes It could be observed that the linear relationship between the number of azo bonds and the rate of removal is maintained. These findings support the occurrence of effective degradation of tested dye skeletons under the action of *S. albidoflavus* 3MGH, implying monitoring of their mineralization [108–110]. The HPLC results could also confirm this. HPLC examination of control dyes and decolorized products yielded distinctively different peak patterns, demonstrating dyes’ decomposition into different molecules. The difference in peak patterns and intensity, as well as retention duration, between pure dyes and dye decolorization products produced by the activity of *S. albidoflavus* 3MGH revealed that the dyes experienced structural modifications. The chromatographic changes in the elution profiles of the decolorized metabolite products confirm the structural changes and complete transformation of the dyes into other forms [75, 111, 112].

Azo dyes are typically composed of aromatic rings connected to one or more azo bonds (―N═N bonds not formed naturally), as well as sulfonated substitutions. Because of their complex structure, azo dyes are fairly stable and resist biological, chemical, and photodegradation as well. Because of structural differences such as chromophore azo group number, auxochrome functionality content, and aromatic system arrangement, the decolorization rate of different synthetic azo dyes varies, and the degradative capacity decreases as the structure becomes more complex [110, 113, 114].

At optimum conditions, the FT-IR spectra of control monoazo (RO 122), diazo (DB 15), and triazo (DB 38) dyes and their metabolite degradation products showed significant changes in the IR spectrum of treated dyes compared to the control spectra, including the elimination, addition, and shift of peaks caused by *S. albidoflavus* 3MGH decolorization. Furthermore, the new peaks at frequencies lower than 1600 cm^−1^ could be the result of azo dye degradation byproducts and the introduction of new functionalities [7, 81]. The chromophore azo-N = N-on aromatic arrangement and of-N = N-stretching in a-substituted structures exhibit peaks at 1561 cm^−1^ with control monoazo dye, 1599 cm^−1^ with control diazo dye, and 1570 cm^−1^ with control triazo dyes [108]. Such peaks have completely disappeared from the spectrum, indicating a rupture at the azo bond site and explaining why decolorization occurs in dye solutions due to the action of *S. albidoflavus* 3MGH. Furthermore, all N-H stretch and bending bands within incubated dyes (mono, di, and tri) shifted to lower frequencies in addition to being broader if compared to control dye spectra. It was stated that a small shift in N-H bending indicates strong color decolorization [115]. It was proposed that the absence and shift of functional groups in dye-treated samples resulted in the development of new compound structures. The removal of noticeable peaks and the development of new peaks in the FTIR spectra of dye-decolorized metabolites demonstrate that *S. albidoflavus* 3MGH successfully biotransformed and mineralized dye samples into simple metabolites [111, 116, 117].

The proposed mechanisms of decolorization of mono (RO 122), di (DB 15), and tri dye (DB 38) azo dyes may be carried out by the initial reductive or oxidative cleavage of azo bonds by the action of different enzymes (laccase and azoreductase) through the conversion of N = N into N_2_ or NH_3_, and afterward into biomass, resulting in colour intensity decreasing or disappearing. Reductive enzymes, such as azoreductase, are believed to cause azo bond deterioration, whereas oxidative enzymes, such as laccase, are believed to cause oxidative cleavage of the resulting amines to smaller intermediates [5, 110, 118]. The GC-MS analysis of the metabolite product profiles of the three dyes supported the proposed degradation mechanism by detecting significant numbers in the proposed fractionation [111, 117, 119].

The breakdown of RO 122 by *S. albidoflavus 3MGH* has been evaluated using GCMS to identify different metabolites. Using mass/charge values, retention time, molecular weight, and chemical structure, a feasible degradation route was identified (Fig. 4; Table 3). The azoreductase ruptured the azo link of the typical dye, generating intermediates 1, 2, and 3. Intermediate 4 developed as a result of the deamination of Intermediate 3. The non-symmetric cleavage of intermediate 4 resulted in the formation of intermediates 5, 6, and 7 (3,7-diamino-4-hydroxynaphthalene-2-sulfonicacid (mw = 254.2, M/Z = 254), RT = 22.68 min.). Furthermore, the non-symmetric cleavage of intermediates 5 and 6 generated cleavage products 8, (8, 2-(4-aminobenzene-1-sulfonyl) ethyl hydrogen sulfate (MW = 281.3, M/Z = 281.0), RT = 26.05 min), and 9, (4-chloro-1,3,5-triazin-2-amine (MW = 130.5, M/Z = 126), RT = 11.78 min). The deamination of fragmentation species 8 and 9 contributed to the emergence of species 10 (2-(benzene sulfonyl) ethyl hydrogen sulfate (MW = 266.2, M/Z = 264.1), RT = 22.61 min.) and 11 (2-chloro-1,3,5-triazine (mw = 115.5, M/Z = 114.03), RT = 4.63 min), respectively. Intermediate 7 is sequentially deaminated, yielding specie 12 (3-amino-4-hydroxynaphthalene-2-sulfonic acid, mw = 239.2, M/Z = 240.2, RT = 25.14 min.) and 13 (4-hydroxynaphthalene-2-sulfonic acid, mw = 224.2, M/Z = 227.1, RT = 26.05 min.).

The molecular weight, mass spectra, retention time, and chemical structure of Direct Blue 15 (DB 15) were utilized for analyzing biodegraded metabolites by *S. albidoflavus* 3MGH using GC-MS. The azo reductase enzyme broke the azo link of the DB 15 dye, resulting in intermediates 1, 2, 3, and 4 (Fig. 5; Table 4). The deamination of intermediates 1 and 2 yielded fermentation spice 5 (naphthalene-2,7-disulfonic acid (mw = 304.4, M/Z = 298.3), RT = 22.8 min), whereas the partial breakdown of the azo link in intermediates 3 and 4 produced pieces 6 (3,3’-dimethoxy[1,1’-biphenyl]-4,4’-diamine (mw = 244.2, M/Z = 252), RT = 26.1 min), 7 (3,3’-dimethoxy [1,1’-biphenyl]. -4-amine (mw = 229.2, M/Z = 227.2), RT = 21.0 min), and 8 (3,3’-dimethoxy-1,1’-biphenyl (mw = 214.2, M/Z = 207), RT = 25.0 min). Compound 9 (2-methoxyaniline (mw = 123, M/Z = 124), RT = 11.2 min) was formed by the symmetric cleavage of intermediate 6, whereas compound 10 (anisole (mw = 108, M/Z = 106.9), RT = 5.3 min) was produced by the symmetric cleavage and deamination of intermediate 7, and 11 (1,1’-biphenyl (mw = 154.2, M/Z = 150), RT = 26.1 min) was produced by the deacylation of intermediate 8.

The oxidoreductive enzymes of *S. albidoflavus* 3MGH degradation of DB 38 were also studied using GCMS to identify metabolites. The activity of such enzymes was responsible for the mineralization of azo dyes through azo bond reduction and/or deamination (successive deamination), which resulted in the formation of many fragmentation intermediates (Fig. 6; Table 5). Many fragmentation intermediates have been identified, including fragmentation products 5 (benzene-1,2,4-triamine (mw = 123, M/Z = 128), RT = 11.2 min), 3, 7, and 12 (aniline (mw = 93, M/Z = 91), RT = 5.3 min), 8, and 17 (benzene (mw = 78.1, M/Z = 74.1), RT = 4.4 min), 10 (1,1’-biphenyl]-4,4’-diamine (mw = 184.2, M/Z = 191.1), RT = 16.6 min.), 13 (3,4,6-triamino-5-hydroxynaphthalene-2,7-disulfonic acid (mw = 349.3, M/Z = 341.3), RT = 27.4 min), 15 (3-amino-5-hydroxynaphthalene-2,7-disulfonic acid (mw = 319.3, M/Z = 318), RT = 28.5 min.), and 16 (3,4-diamino-5-ydroxynaphthalene-2,7-disulfonic acid (mw = 334.3, M/Z = 337.3), RT = 28.1 min.). So, the formation of intermediates with a lower molecular weight suggests that the enzymes from *S. albidoflavus* 3MGH broke down the dyes, which led to its mineralization.

## Conclusion

Disposal of dyeing effluents from the textile industry poses a significant challenge to sustainable environmental growth. Different physicochemical methods and chemical processes are employed to treat dye-contaminated wastewater. However, these techniques often suffer from drawbacks such as high costs, significant energy requirements, limited adaptability to varying dye concentrations, and the generation of secondary pollutants. Actinobacteria are emerging as promising alternatives for the treatment of wastewater containing diverse synthetic dyes, offering a safe and effective means of disposal in the environment. In this study, an actinobacterial isolate from Egypt’s polluted soil with textile waste was identified as *S. albidoflavus 3MGH*. It showed strong color-removing abilities against three azo dyes: RO 122, DB 15, and DB 38. The initial variables (pH, temperature, dye concentration, incubation time, C-sources, and N-sources) were found to play a crucial role in the efficiency of the decolorization process. To analyze the biotransformation process of dyes into degraded metabolites, FT-IR, high-performance liquid chromatography, and gas chromatography-mass spectrometry were used. GC-MS analysis was used to confirm the bio-decolorization of all dyes and allowed for the identification of the intermediate products of the tested dyes. Future research is required to study enzyme activity during dye degradation and evaluate metabolite toxicity to further understand the efficient azo dye bio-removal potential of *S. albidoflavus* 3MGH.

### Supplementary Information

Supplementary Material 1.

## Data Availability

All the data are available in the manuscript. The 16 S rRNA sequence of the most potent azo dye degrading Streptomycesisolate S. albidoflavus 3MGH was deposited into Genbank with accession #MW185782. https://www.ncbi.nlm.nih.gov/nuccore/MW185782.1/.
