# Supplementary material for: Exploring the decolorization efficiency and biodegradation mechanisms of different functional textile azo dyes by Streptomyces albidoflavus 3MGH
Source: BMC Microbiol. 2024 Jun 14;24:210. doi: 10.1186/s12866-024-03347-9 (PMC11179346; doi:10.1186/s12866-024-03347-9)
Supplement: Supplementary file 1 — Supplementary Material 1. [file 12866_2024_3347_MOESM1_ESM.docx]

**Supplementary Material**.

**Exploring the Decolorization Efficiency and Biodegradation Mechanisms of Different Functional Textile Azo Dyes by *Streptomyces albidoflavus* 3MGH**

Mohamed E. El Awady^1,^ Fatma N. El-Shall^2^, Ghada E. Mohamed^3^, Ahmed M. Abd-Elaziz^4^, Mohamed O. Abdel-Monem^3*^ and Mervat G. Hassan^3^

| **Supplementary table 1.** Chemical structure of selected azo dyes used in study. | | |
| --- | --- | --- |
| C.I.name | Functionality | Molecular Formula, Molecular Weight, and Chemical Structure |
| Reactive [Orange](http://www.worlddyevariety.com/?s=Orange&submit=Search) 122 | Mono | C_31_H_20_ClN_7_Na_4_O_16_S_5_ 1034.27 g·mol^−1^ |
| Direct Blue 15 | Di | C_34_H_24_N_6_Na_4_O_16_S_4_ 992.79 g·mol^−1^   |
| Direct Black 38 | Tri | C_34_H_25_N_9_Na_2_O_7_S_2_ 781.73 g.mol^-1^   |


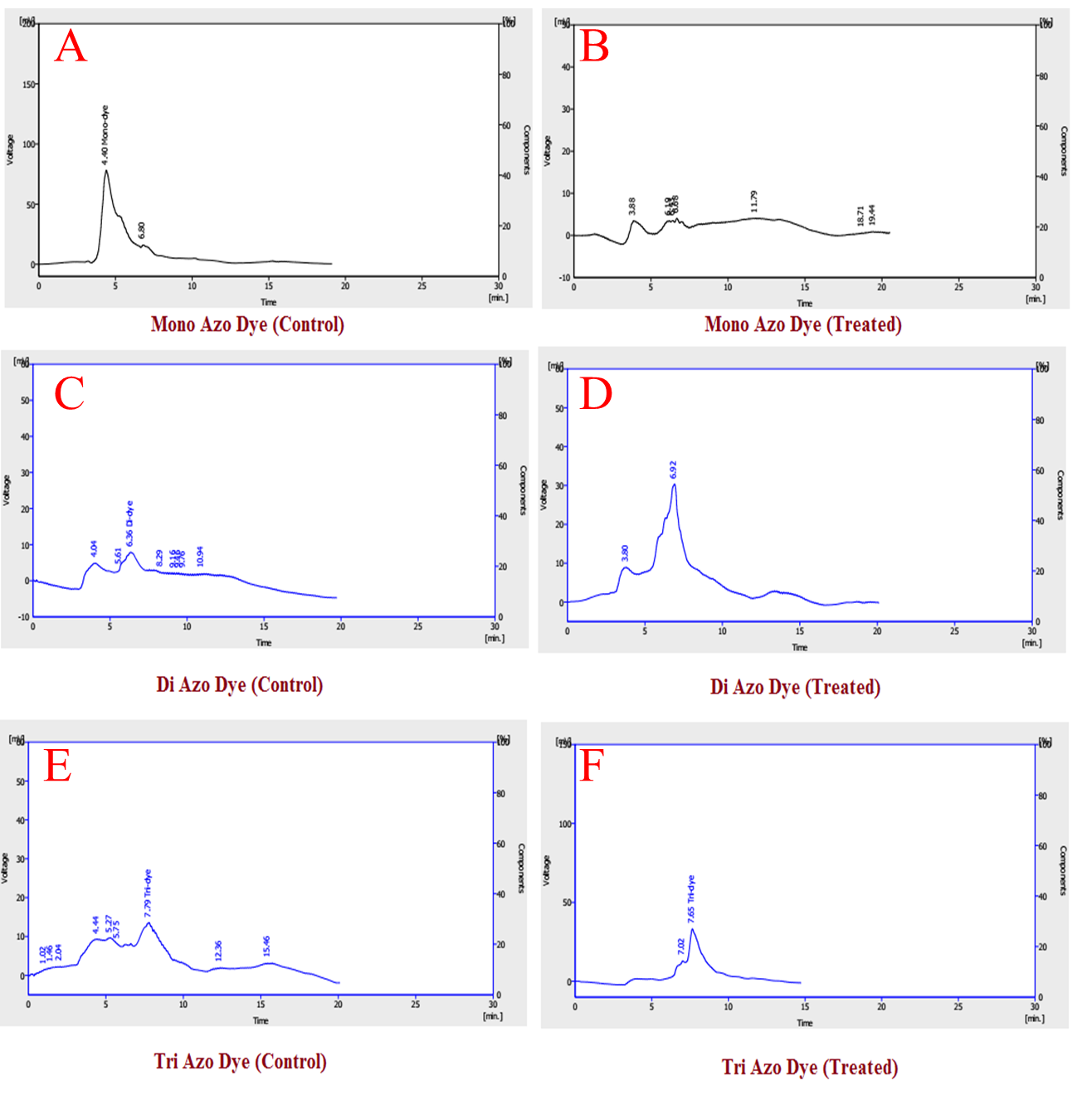


**Supplementary figure 1:** HPLC Chromatogram Profiles of Mono Azo Dye (RO 122) (**A&B**), Di Azo Dye (DB 15) (**C&D**), and Tri Azo Dye (DB 38) (**E&F**) before and after treatment with *S. albidoflavus* 3MGH.
